# Between continuities and innovations: what CSP has in store for 2026

**DOI:** 10.1590/0102-311XEN227925

**Published:** 2026-01-09

**Authors:** Luciana Correia Alves, Marilia Sá Carvalho, Luciana Dias de Lima

**Affiliations:** 1 Instituto de Filosofia e Ciências Humanas, Universidade Estadual de Campinas, Campinas, Brasil.; 2 Programa de Computação Científica, Fundação Oswaldo Cruz, Rio de Janeiro, Brasil.; 3 Escola Nacional de Saúde Pública Sergio Arouca, Fundação Oswaldo Cruz, Rio de Janeiro, Brasil.

The end of an academic and scientific year is always a moment of reflection - an opportunity to evaluate the path taken, improve practices and rethink choices. Populations are dynamic, thoughts are transformed, and society reveals new trends. In the field of scientific publication and communication, it is no different: we are facing a permanent transformation process.

Over its 40 years, CSP has shown itself to be a journal attentive to its time, with an editorial board capable of recognizing the necessary changes, rethinking its practices, opening new fronts and, when necessary, redefining its paths ^1^. This capacity for renewal is a hallmark that has accompanied CSP since its creation.

From 1985 until today, CSP has published its issues as fascicles, a classic format that has featured in the formation of several national and international journals over time. During this period, each issue represented more than a set of articles. It marked the emergence of new editorial structures and the increased importance of sections, introducing innovative formats for scientific publication. CSP’s regular periodicity allowed the consolidation of an editorial identity, since the publication of each issue is necessarily accompanied by an editorial for its opening. In addition, publication in installments maintains a constant dialogue with the scientific community and structures a calendar of publications that is repeated and ends up creating an expected flow of research communication for authors and readers.

In CSP, the format of fascicles played a historical and fundamental role. It favored the organization of volumes, indexing in databases and the visibility of the themes of each issue. CSP fascicles also served as milestones: they opened space for thematic supplements, tributes, and editorial reflections that helped to build our scientific memory.

However, scientific communication has been rapidly transforming ^2^. Open access, the internationalization of publications ^3^ and the intensive use of digital platforms have brought new demands to academic journals. In this context, in 2019, SciELO ^4^ began to encourage the continuous publication model as a natural response to the contemporary challenges of knowledge dissemination. This format eliminates the need to wait for the composition of a complete issue to publish new articles, allowing greater agility in publication ^4^.

In 2026, CSP will adopt the continuous publication model in a single annual volume, which marks a new phase for the journal - more agile, accessible and in line with international best practices in scientific publication. Like any change, it will require some adjustments in the editorial routine and internal workings, but we understand that this transition will bring significant gains, especially for authors.

Reducing the interval between approval and publication of manuscripts, and ensuring greater transparency in the dissemination of scientific knowledge is part of CSP’s editorial mission ^5^. Time is one of the great challenges in scientific publishing nowadays and the continuous publication model can contribute to reducing this interval. Along with the change to this model, we intend to expand our scientific dissemination strategies, increasing the reach and social impact of the articles.

Within this continuous publication format, CSP will continue to publish its traditional sections - such as thematic space, debate, thematic supplements and editorials - which constitute one of the main marks of its current format and which the journal and the publishers will not give up. The editorials, in particular, express editorial policies, political positions and structural changes, elements that inform CSP’s identity. In the same direction, the cover photos - which since 1996 have been part of CSP’s visual identity and scientific communication strategy - will continue. Each year, these historical, journalistic, or documentary images, selected in dialogue with the central theme, seek to visually translate the forms of societal organization and the living and health conditions of the population ^1^. In the new continuous publication format, the covers will gain greater frequency and will be adapted to the new editorial dynamics, also associating themselves with the moments when the publishers publish editorials on new journal guidelines.

In 2025, CSP took an important step in its editorial policy by implementing another structural change in line with the Open Science principles ^6^, moving towards a more collaborative and transparent scientific communication model. Since August, the journal has been publishing the name of the Editor or Associate Editor responsible for each published article and, in 2026, it will also inform the names of reviewers who agree to have their identity revealed. We believe that the disclosure of information regarding the peer review process should be done gradually and responsibly, seeking to strengthen the trust of all actors involved in the evaluation process.

In October, CSP began to incorporate, in the OJS system, information on research data availability, including extraction codes, analyses, results, and links to repositories. Authors must indicate the option that best corresponds to the data available in their article, and this information will be included in the final version published. This is a relevant implemented initiative which reinforces CSP’s commitment to the transparency and openness of scientific data.

CSP’s theme in 2026 will be “Brazilianness”. CSP has always been concerned with diversity and thematic plurality in its publication. And next year we will seek to reflect on the diversity that marks Brazil’s scientific production. Brazilian science is made up of different perspectives, regions, and trajectories, and this plurality is one of its greatest strengths. Recognizing Brazilianness is valuing the knowledge that is born from cultural, social, and territorial diversity and validates the commitment to an open, inclusive science that is connected to the country’s challenges. The colors representing us on next year’s cover are those of the Brazilian flag and will symbolize this movement, with emphasis on yellow - a light that points to the future and inspires the construction of more equitable scientific and social paths, reaffirming our sovereignty.

All these changes confirm CSP’s mission to promote the circulation of ideas and research results in an increasingly broad, ethical and innovative manner.

Happy 2026!
