# Supplementary material for: Between continuities and innovations: what CSP has in store for 2026
Source: Cad Saude Publica. 2026 Jan 9;41(12):e00227925. doi: 10.1590/0102-311XEN227925 (PMC12799127; doi:10.1590/0102-311XEN227925)
Supplement: Translation

# Translation: Entre continuidades e inovações: o que CSP reserva para 2026

**DOI:** 10.1590/0102-311XPT227925

**Authors:** Luciana Correia Alves, Marilia Sá Carvalho, Luciana Dias de Lima

**Affiliations:** 1 Instituto de Filosofia e Ciências Humanas, Universidade Estadual de Campinas, Campinas, Brasil.; 2 Programa de Computação Científica, Fundação Oswaldo Cruz, Rio de Janeiro, Brasil.; 3 Escola Nacional de Saúde Pública Sergio Arouca, Fundação Oswaldo Cruz, Rio de Janeiro, Brasil.

O encerramento de um ano acadêmico e científico é sempre um momento de reflexão, uma oportunidade para avaliar o caminho percorrido, aprimorar práticas e repensar escolhas. As populações são dinâmicas, os pensamentos se transformam e a sociedade revela novas tendências. No campo da publicação e da comunicação científica, não é diferente: estamos diante de um processo permanente de mudança.

Ao longo de seus 40 anos de existência, CSP tem se mostrado um periódico atento a seu tempo, com um corpo editorial capaz de reconhecer as alterações necessárias, repensar suas práticas, abrir novas frentes e, quando preciso, redefinir seus caminhos ^1^. Essa capacidade de renovação é uma marca que acompanha CSP desde sua criação.

De 1985 até hoje, CSP tem publicado seus números na forma de fascículos, um modelo clássico e que fez parte da conformação de diversos periódicos nacionais e internacionais ao longo do tempo. Nesse período, cada fascículo representou mais do que um conjunto de artigos. Marcou o surgimento de novas estruturas editoriais e a valorização das seções, introduzindo formatos inovadores para a publicação científica. A periodicidade regular permitiu consolidar uma identidade editorial, uma vez que a publicação de cada fascículo é acompanhada necessariamente de um editorial para sua abertura. Ainda, a publicação em fascículos mantém um diálogo constante com a comunidade científica e estrutura um calendário de publicações que se repete e que acaba criando um fluxo esperado da comunicação da pesquisa para autores e leitores.

Em CSP, o formato de fascículos cumpriu um papel histórico e fundamental. Ele favoreceu a organização dos volumes, a indexação nas bases de dados e a visibilidade dos temas de cada número. Os fascículos em CSP também serviram como marcos: abriram espaço para suplementos temáticos, homenagens e reflexões editoriais que ajudaram a construir a nossa memória científica.

Entretanto, a comunicação científica tem se transformado de maneira acelerada ^2^. O acesso aberto, a internacionalização das publicações ^3^ e o uso intensivo de plataformas digitais trouxeram novas demandas para os periódicos acadêmicos. Nesse contexto, em 2019, a SciELO ^4^ passa a incentivar o modelo de publicação em fluxo contínuo como uma resposta natural aos desafios contemporâneos da disseminação do conhecimento. Esse formato elimina a necessidade de aguardar a composição de um fascículo completo para a publicação de novos artigos, permitindo maior agilidade na publicação ^4^.

Em 2026, CSP passará a adotar o modelo de publicação contínua em volume único anual, que marca uma nova fase para a revista - mais ágil, acessível e alinhada às boas práticas internacionais de publicação científica. Como toda mudança, exigirá alguns ajustes na rotina editorial e no funcionamento interno, mas entendemos que essa transição trará ganhos significativos, especialmente para os autores.

Reduzir o intervalo entre a aprovação e a divulgação dos manuscritos, bem como garantir maior transparência na disseminação do conhecimento científico integra a missão editorial de CSP ^5^. O tempo é um dos grandes desafios na publicação científica atualmente e o modelo de fluxo contínuo pode contribuir para a diminuição desse intervalo. Paralelamente à mudança para o modelo de publicação contínua, pretendemos ampliar nossas estratégias de divulgação científica, ampliando o alcance e o impacto social dos artigos.

Dentro desse formato de fluxo contínuo, CSP continuará a publicar suas seções tradicionais - como o espaço temático, o debate, os suplementos temáticos e os editoriais -, que constituem uma das principais marcas de seu formato atual e das quais a revista e as editoras não abrem mão. Os editoriais, em particular, expressam as políticas editoriais, os posicionamentos políticos e as mudanças estruturais, elementos que fazem parte da identidade de CSP. Na mesma direção, as fotos de capa - que desde 1996 fazem parte da identidade visual e da estratégia de comunicação científica de CSP - continuarão presentes. A cada ano, essas imagens históricas, jornalísticas ou documentais, selecionadas em diálogo com o tema central, buscam traduzir visualmente as formas de organização da sociedade e as condições de vida e saúde da população ^1^. No novo formato de publicação contínua, as capas ganharão maior frequência e serão adaptadas à nova dinâmica editorial, associando-se também aos momentos em que as editoras publicarem editoriais sobre novas diretrizes da revista.

Em 2025, CSP deu um importante passo em sua política editorial ao implementar mais uma mudança estrutural alinhada aos princípios da Ciência Aberta ^6^, avançando para um modelo de comunicação científica mais colaborativo e transparente. Desde agosto, a revista passou a divulgar o nome do Editor ou da Editora Associado(a) responsável por cada artigo publicado e, em 2026, também passará a informar os nomes dos revisores que aceitarem ter sua identidade revelada. Acreditamos que a abertura de informações referentes ao processo de revisão por pares deve ser feita de forma gradual e responsável, buscando fortalecer a confiança de todos os atores envolvidos no processo de avaliação.

Em outubro, CSP passou a incorporar, no sistema OJS, informações sobre a disponibilidade dos dados de pesquisa, incluindo códigos de extração, análises, resultados e *links* para repositórios. Os autores devem indicar a opção que melhor corresponda aos dados disponíveis em seu artigo, e essa informação passará a constar na versão final da publicação. Trata-se de uma iniciativa relevante, implementada, que reforça o compromisso de CSP com a transparência e a abertura dos dados científicos.

O tema de CSP em 2026 será “brasilidades”. CSP sempre teve uma preocupação com a diversidade e pluralidade temática em sua publicação. E, no próximo ano, buscaremos refletir sobre a diversidade que marca a produção científica do país. A ciência brasileira é feita de diferentes perspectivas, regiões e trajetórias, e essa pluralidade é uma de suas maiores forças. Reconhecer as brasilidades é valorizar o conhecimento que nasce da diversidade cultural, social e territorial, além de validar o compromisso com uma ciência aberta, inclusiva e conectada com os desafios do país. As cores que nos representam na capa do próximo ano são as da bandeira do Brasil e irão simbolizar esse movimento, com destaque para o amarelo - luz que aponta para o futuro e inspira a construção de caminhos científicos e sociais mais equitativos, reafirmando nossa soberania.

Todas essas mudanças confirmam a missão de CSP de promover a circulação de ideias e resultados de pesquisa de forma cada vez mais ampla, ética e inovadora.

Feliz 2026!

# Translation: Entre continuidades e innovaciones: lo que CSP reserva para 2026

**DOI:** 10.1590/0102-311XES227925

**Authors:** Luciana Correia Alves, Marilia Sá Carvalho, Luciana Dias de Lima

**Affiliations:** 1 Instituto de Filosofia e Ciências Humanas, Universidade Estadual de Campinas, Campinas, Brasil.; 2 Programa de Computação Científica, Fundação Oswaldo Cruz, Rio de Janeiro, Brasil.; 3 Escola Nacional de Saúde Pública Sergio Arouca, Fundação Oswaldo Cruz, Rio de Janeiro, Brasil.

El cierre de un año académico y científico es siempre un momento de reflexión, una oportunidad para evaluar el camino recorrido, mejorar las prácticas y reconsiderar las decisiones tomadas. Las poblaciones son dinámicas, el pensamiento cambia y la sociedad revela nuevas tendencias. En el campo de la publicación y de la comunicación científica no es diferente: nos enfrentamos a un proceso de cambio permanente.

A lo largo de sus 40 años de existencia, CSP ha demostrado ser atenta a su tiempo, con un equipo editorial capaz de reconocer los cambios necesarios, replantearse sus prácticas, abrir nuevos frentes y, cuando es necesario, redefinir sus rumbos ^1^. Esta capacidad de renovación es un sello distintivo que la ha acompañado desde su creación.

Desde 1985 hasta hoy, CSP ha publicado sus números en forma de fascículos, un modelo clásico que ha formado parte de la estructura de diversas revistas nacionales e internacionales a lo largo del tiempo. En este período, cada fascículo representó más que un conjunto de artículos. Marcó el surgimiento de nuevas estructuras editoriales y la valoración de las secciones, introduciendo formatos innovadores para la publicación científica. La periodicidad regular ha permitido consolidar una identidad editorial, ya que la publicación de cada fascículo va acompañada necesariamente de un editorial para su apertura. Además, la publicación en fascículos mantiene un diálogo constante con la comunidad científica y estructura un calendario de publicaciones que se repite y que acaba creando un flujo esperado de comunicación de la investigación para autores y lectores.

En CSP, el formato de fascículos desempeñó un papel histórico y fundamental. Favoreció la organización de los volúmenes, la indexación en las bases de datos y la visibilidad de los temas de cada número. Los fascículos en CSP también sirvieron como hitos: abrieron espacio para suplementos temáticos, homenajes y reflexiones editoriales que ayudaron a construir nuestra memoria científica.

Sin embargo, la comunicación científica ha cambiado rápidamente ^2^. El acceso abierto, la internacionalización de las publicaciones ^3^ y el uso intensivo de plataformas digitales han traído nuevas exigencias para las revistas académicas. En este contexto, en 2019, SciELO ^4^ pasó a fomentar el modelo de publicación en flujo continuo como respuesta natural a los retos contemporáneos de la difusión del conocimiento. Este formato elimina la necesidad de esperar a que se complete un fascículo para publicar nuevos artículos, lo que permite una mayor agilidad en la publicación ^4^.

En 2026, CSP adoptará el modelo de publicación continua en un único volumen anual, lo que marca una nueva etapa: más ágil, accesible y alineada con las buenas prácticas internacionales de publicación científica. Como todo cambio, requerirá algunos ajustes en la rutina editorial y en el funcionamiento interno, pero entendemos que esta transición traerá beneficios significativos, especialmente para los autores.

Reducir el intervalo entre la aprobación y la divulgación de los manuscritos, así como garantizar una mayor transparencia en la difusión del conocimiento científico, forma parte de la misión editorial de CSP ^5^. El tiempo es uno de los grandes retos de la publicación científica en la actualidad y el modelo de flujo continuo puede contribuir a reducir este intervalo. Paralelamente al cambio al modelo de publicación continua, pretendemos ampliar nuestras estrategias de divulgación científica, ampliando el alcance y el impacto social de los artículos.

Dentro de este formato de flujo continuo, CSP seguirá publicando sus secciones tradicionales -como el espacio temático, el debate, los suplementos temáticos y los editoriales-, que constituyen una de las principales características de su formato actual y a las que la revista y las editoriales no abandonarán. Los editoriales, en particular, expresan las políticas editoriales, las posiciones políticas y los cambios estructurales, elementos que forman parte de la identidad de la revista. En la misma línea, las fotos de portada, que desde 1996 forman parte de la identidad visual y la estrategia de comunicación científica de CSP, seguirán estando presentes. Cada año, estas imágenes históricas, periodísticas o documentales, seleccionadas en diálogo con el tema central, buscan traducir visualmente las formas de organización de la sociedad y las condiciones de vida y salud de la población ^1^. En el nuevo formato de publicación continua, las portadas se harán más frecuentes y se adaptarán a la nueva dinámica editorial, asociándose también a los momentos en que las editoriales publiquen editoriales sobre las nuevas directrices de la revista.

En 2025, CSP dio un paso importante en su política editorial al implementar otro cambio estructural alineado con los principios de la Ciencia Abierta ^6^, avanzando hacia un modelo de comunicación científica más colaborativo y transparente. Desde agosto, la revista comenzó a divulgar el nombre del editor o editora asociado responsable de cada artículo publicado y, en 2026, también comenzará a informar los nombres de los revisores que acepten revelar su identidad. Creemos que la divulgación de información relativa al proceso de revisión por pares debe realizarse de forma gradual y responsable, con el fin de reforzar la confianza de todos los actores involucrados en el proceso de evaluación.

En octubre, CSP comenzó a incorporar en el sistema OJS información sobre la disponibilidad de los datos de investigación, incluyendo códigos de extracción, análisis, resultados y enlaces a repositorios. Los autores deben indicar la opción que mejor se ajuste a los datos disponibles en su artículo, y esta información se incluirá en la versión final de la publicación. Se trata de una iniciativa relevante, ya implementada, que refuerza el compromiso de la revista con la transparencia y la apertura de los datos científicos.

El tema de CSP en 2026 será “brasileñidades”. La revista siempre se ha preocupado por la diversidad y la pluralidad temática en sus publicaciones. Y el próximo año trataremos de reflexionar sobre la diversidad que caracteriza la producción científica del país. La ciencia brasileña se compone de diferentes perspectivas, regiones y trayectorias, y esa pluralidad es una de sus mayores fortalezas. Reconocer la brasileñidad es valorar el conocimiento que nace de la diversidad cultural, social y territorial, y valida el compromiso con una ciencia abierta, inclusiva y conectada con los retos del país. Los colores que nos representan en la portada del próximo año son los de la bandera de Brasil y simbolizarán este movimiento, con especial destaque para el amarillo, luz que apunta hacia el futuro e inspira la construcción de caminos científicos y sociales más equitativos, reafirmando nuestra soberanía.

Todos estos cambios confirman la misión de CSP de promover la circulación de ideas y resultados de investigación de una manera cada vez más amplia, ética e innovadora.

¡Feliz 2026!
